# Evaluation of an automatic article selection method for timelier updates of the Comet Core Outcome Set database

**DOI:** 10.1093/database/baz109

**Published:** 2019-11-07

**Authors:** Christopher R Norman, Elizabeth Gargon, Mariska M G Leeflang, Aurélie Névéol, Paula R Williamson

**Affiliations:** 1 LIMSI, CNRS, Université Paris-Saclay, Bât 507, rue du Belvédère, Campus Universitaire, F-91405 Orsay; 2 Amsterdam Public Health, Amsterdam Umc, University of Amsterdam, Meibergdreef 9, 1105 az, Amsterdam, the Netherlands; 3 MRC NWHMTR, Department of Biostatistics, University of Liverpool, Liverpool, UK

## Abstract

Curated databases of scientific literature play an important role in helping researchers find relevant literature, but populating such databases is a labour intensive and time-consuming process. One such database is the freely accessible Comet Core Outcome Set database, which was originally populated using manual screening in an annually updated systematic review. In order to reduce the workload and facilitate more timely updates we are evaluating machine learning methods to reduce the number of references needed to screen. In this study we have evaluated a machine learning approach based on logistic regression to automatically rank the candidate articles. Data from the original systematic review and its four first review updates were used to train the model and evaluate performance. We estimated that using automatic screening would yield a workload reduction of at least 75% while keeping the number of missed references around 2%. We judged this to be an acceptable trade-off for this systematic review, and the method is now being used for the next round of the Comet database update.

## Introduction

A wealth of biomedical information is buried in the free text of scientific publications. Curated databases play a major role in helping researchers and clinicians access this data, by selecting articles and specific facts of interest in the subfield of biomedicine they address (12, 17).

One such database is maintained by the Core Outcome Measures in Effectiveness Trials (COMET) Initiative, which aims to improve the usefulness of outcomes in research and help tackle problems such as outcome reporting bias, inconsistency and lack of importance or relevance of outcomes to patients. These problems are being addressed through the development and use of core outcome sets (COS). A COS is an agreed standardized set of outcomes that should be measured and reported, as a minimum, in all trials for a specific clinical area (28). COMET facilitates the development and application of COS, by bringing relevant material together and thus making it more accessible. Since 2011, COMET has maintained a public repository of studies relevant to the development of COS (The COMET database, http://www.comet-initiative.org/studies/search). The database was originally populated through completion of a systematic review (8), which is annually updated to include all published COS, currently up to and including December 2017 (5, 7, 10, 11).

The database is an integral resource not only to the development of COS, but also to the uptake of COS in research and in the avoidance of unnecessary duplication and waste of scarce resources (7).

A survey demonstrated that the database is also used by a variety of other users in addition to COS developers, including clinical trialists, systematic reviewers, auditors, guideline developers and funders (11).

Relevant studies are added to the database as they are found, but the annual update to the systematic review is necessary to ensure completeness.

A two-stage process is employed to screen records and identify relevant studies. Titles and abstracts are read to assess eligibility of studies for inclusion in the review (stage 1). Full texts of potentially relevant articles are obtained to assess for inclusion (stage 2). Studies are eligible for inclusion if they have applied methodology for determining which outcomes or outcome domains should be measured in clinical trials or other forms of health research. Relevant studies therefore describe the development of a COS, regardless of any restrictions by age, health condition or setting. The inclusion and exclusion criteria are described in more detail in the original systematic review (8).

We encounter challenges in undertaking this comprehensive approach, such as the variability in free text terms and index terms used for COS development, further confounded by the absence of a specific index term or Medical Subject Heading (MeSH) main heading for this study type (9).

The term ‘core outcome set’ has not been commonly used until recently, and is still not consistently applied with many variations employed to describe this type of study (e.g. core domain set, core measurement set, minimum outcome set), and they do not appear to be categorized consistently across different databases. Furthermore, no single database specializes in this type of methodological research and it is likely to be found across a wide range of literature. Finally, the search is not limited by condition or disease, setting, study type or intervention. A direct consequence of these challenges is the work involved in manually screening a large number of records on an annual basis. The latest update (7) took $7$ months from running the searches in March 2018 to submission of the manuscript in early October 2018, and involved five reviewers. It is a labour intensive review and therefore costly to keep this up to date. With the need to update this annually, a balance needs to be struck between managing this workload and the likelihood that all eligible studies will be identified. The addition of a new index term or MeSH heading to identify COS is unlikely at this time, so it is imperative that we explore alternative routes in an attempt to streamline this process.

## Screening automation in systematic reviews

Automation has great potential to make systematic reviews quicker and cheaper (2, 26). Recent advances in text mining, natural language processing and machine learning have demonstrated that tasks within the systematic review process can be automated or assisted by automation. Possible tasks include screening of titles and abstracts, sourcing full texts and data extraction. Automation to assist the screening process is of particular interest in these systematic review updates due to the high number of hits retrieved in the annual searches.

Using automated methods to prioritize the order in which references are screened is considered safe for use in prospective systematic reviews, but using cut-off values to eliminate studies automatically is not recommended practice (21). A wide range of methods have been proposed for this kind of screening prioritization, including Support Vector Machines, Naive Bayes, Voting Perceptrons, LAMBDA-Mart, Decision Trees, EvolutionalSVM, WAODE, kNN, Rocchia, hypernym relations, ontologies, Generalized Linear Models, Convolutional Neural Networks, Gradient Boosting Machines, Random Indexing and Random Forests (14, 16, 21, 24). Several screening prioritization systems are publicly available, including EPPI-Reviewer, Abstrackr, SWIFT-Review, Rayyan, Colandr and RobotAnalyst (13, 16, 23, 25, 27).

Comparing the relative performance of different methods is difficult since most methods have been evaluated on different datasets, under different settings, and with different metrics. There have been attempts to compare previous methods by replicating reported methods on the same datasets, but the replication of published methods is often difficult or impossible due to insufficient reporting (20). Performance varies depending on included study type (e.g. randomized control trial, diagnostic study), clinical setting, research question, number of candidate references, etc., and it is therefore seldom possible to extrapolate performance on new, untested systematic reviews from previous experiments.

Conventional screening automation is based on learning-to-rank, an information retrieval approach that uses machine learning or statistics to learn a ranking model from existing training data (6). In the original formulation, a model is trained to estimate the relevance of each candidate reference (pointwise learning), and the references can then be presented to the screeners in descending order of estimated relevance. This is a form of probability regression and has been implemented using a multitude of methods from machine learning and statistics (21). However, in a ranking scenario it may be better to minimize the number of inversions, the number of pairs such that a relevant reference occurs after a non-relevant one, rather than the estimated probability score. This is known as ordinal regression, and can be done using machine learning methods by training on pairs of references (pairwise training) or on an entire list of references (listwise training) (3).

## Material and methods

To develop and evaluate our method, we used the results from the manual screening conducted in the systematic review, and its four annual updates (Table 1) (5, 7, 8, 10, 11).

**Table 1:** Review update

	Original systematic review			1			2			3			4		
	Abs.	Ded.	All	Abs.	Ded.	All	Abs.	Ded.	All	Abs.	Ded.	All	Abs.	Ded.	All
All (A)	24384	27375	28371	4587	4226	4980	3785	3984	4090	4043	4226	4406	4963	5140	5140
Maybe (M)	2220	2290	2346	297	414	429	187	238	248	370	492	519	455	514	514
Yes (Y)	195	217	220	29	30	31	22	24	24	12	15	16	68	70	70

### Data preprocessing

Before experimenting on the data we preprocessed it to ensure that it conforms to a few standard constraints necessary for the experiments to work as intended.

In particular, training and evaluating on the same data points would overestimate the performance, and we therefore preprocess the data so that the training and evaluation sets do not overlap.

Furthermore, removing duplicate data points means that each data point is counted only once in the evaluation of the results.

References may have two publication dates in their bibliographic records, once when they are published online (ahead of print), and once when they appear in the printed journal. When duplicate publication dates span review iterations, references may therefore occasionally be considered in two consecutive review iterations. In the manual screening for the COMET systematic review such duplicate references were screened in both updates they appeared in. Removing these would have required more work than simply screening them, and screening the same references twice will only provide an extra check and will not be detrimental to the review.

**Figure 1:**
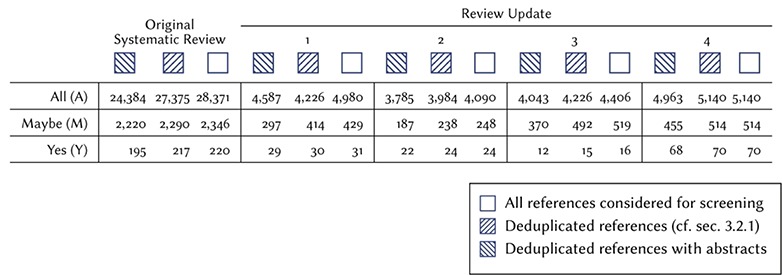
Description of the data used in this study, resulting from the original systematic review, and its review updates. We use the following shorthand for the different stages of the screening process: all (A): references initially identified through the database search. Maybe (M): references provisionally included based on title and abstract, but not yet screened based on full-text. Yes (Y): references judged relevant based on full-text and included in the COMET database.

**Figure 2:**
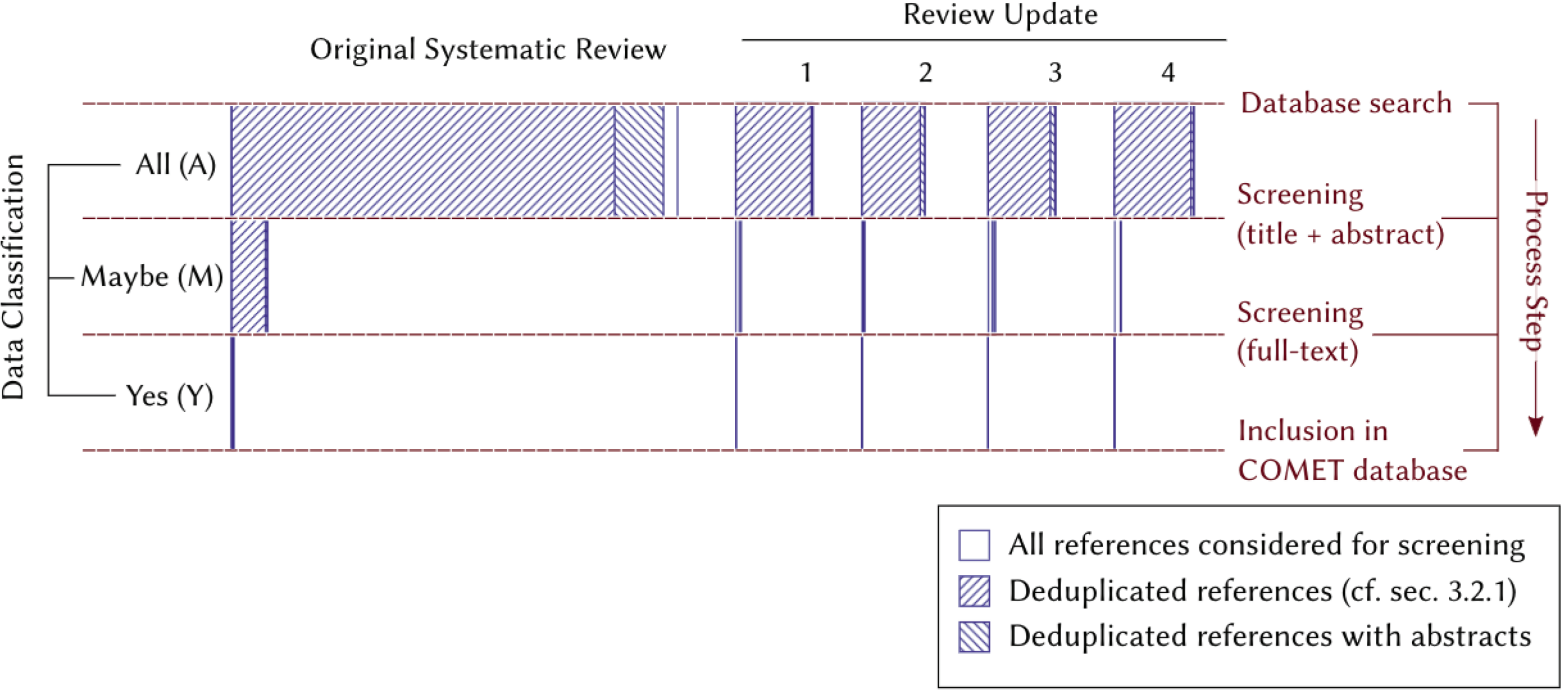
Visual diagram of the flow of references during the manual screening process in the systematic review and the four review updates. The width of each bar corresponds to their respective numbers in Table 1.

For this reason, 1026 references in the original systematic review were re-screened in update 1, 103 references in update 1 were re-screened in update 2, 95 references in update 2 were re-screened in update 3 and 180 references in update 3 were re-screened in update 4. In total, 5 out of 354 included references were considered in at least two review updates (see set Y, Figures 1 and 2).

We opted to remove these duplicate references from the training set, rather than the test set, to mirror how these were handled in the systematic review. In practical terms, this means the model will always judge re-examined references without being biased by (or simply repeating) the judgement shown in the previous review update.

### Document ranking method

To rank references, we used a static ranking method that we have described previously (19) and which performed in the top tier of methods evaluated in the CLEF eHealth international challenge dedicated to Technologically Assisted Reviews in Empirical Medicine (14, 15), and which compared particularly favourably to other models not relying on active learning (similarly to the setup used in this study).

Similar approaches have been used effectively in recent prospective reviews (1, 18, 23). For instance, a recently published, large-scale systematic review on animal studies by Bannach-Brown and colleagues (1) used active learning with similar training, except using SVM instead of logistic regression. Similarly, another recently published living network meta analysis by Lerner and colleagues (18) used the same approach, except with word embeddings instead of *n*-grams.

We evaluated the model on each review update by examining how early it would have ranked the included references (set Y deduplicated in Figures 1 and 2).

**Figure 3:**
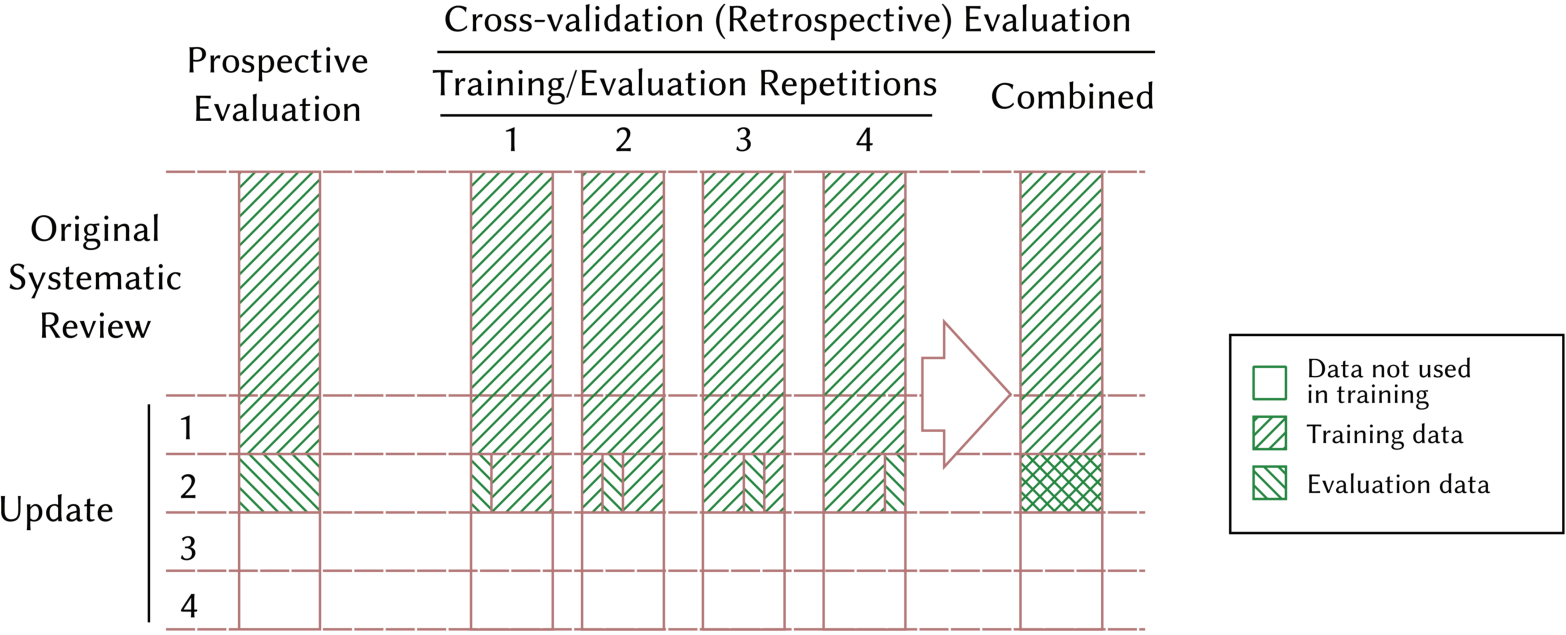
Illustration of our prospective and retrospective (cross-validation) experimental setups when evaluating the performance of the model on update 2. For simplicity we illustrate using $4$-folds instead of $10$. This setup allows us to also use update 2 as training data when evaluating on update 2, while avoid training and evaluating on the same individual references.

We performed two sets of experiments. First, we performed a simulated prospective evaluation (The evaluation is prospective for the model, since it is not allowed to see the future data in the experiments. This study as a whole is still retrospective since the ‘future’ data already existed when we performed the experiments.) on each of the four review updates. In each of these four experiments, we trained a model on the deduplicated data from the prior review iterations. Thus, we for instance trained the model on the data from the original systematic review and updates 1 and 2 when we evaluated the model on the update 3. Second, we evaluated on the original systematic review and on each of the four review updates by adding cross-validated data to the data from previous review data (see Figure 3).

For instance, for update 2 we split the references into $10$ random sets. For each of these $10$ sets we trained a model on the data from other nine sets in addition to the original review and update 1, and let the model calculate scores for each reference in the set held out from training. We then constructed a single ranking by merging the $10$ sets and ranking this set by the score assigned to each reference. We performed these experiments because we suspected that we might get better performance when adding data from the same review update, either because of conceptual drift (4) or simply because of the increase in the amount of training data. This setup also allowed us to evaluate the performance on the original systematic review update, which contains more data than the four review updates combined.

Abstracts were not available for all references, and we therefore performed two sets of experiments to determine to what extent abstracts are necessary for judgement. First, we performed one set of experiments where we trained and evaluated a model using information from the titles and abstracts. In this setup we excluded references for which abstracts were missing. Second, we performed one set of experiment where we trained and evaluated a model using information only from the titles. In this setup we used titles from all references for training (including references with abstracts) and evaluated it on references without abstracts. Second, we performed one set of experiments where we evaluated the model using information only from the titles. We used the same model as in previously, trained on titles and abstracts from all references in the training sets, as well as a model trained only on titles.

### Implementation

We constructed a ranker by extracting bag-of-$n$-grams ($n \leq 5$) over words in the titles and abstracts. We used both tf-idf scores and binary features, in both stemmed and unstemmed form. In previous experiments, 4-grams and 5-grams have yielded consistent but very minor performance improvements, and could have been omitted without substantially decreasing performance. However, the stochastic gradient descent training does not take substantially longer to train on higher order n-grams, and we prefer that unhelpful features be discarded by the training, based on the data. We did not use feature selection, or dimension reduction.

We used the implementation of logistic regression in sklearn (22) using version 0.20.2 trained using stochastic gradient descent, i.e. the SGDClassifier trained using log loss. We trained the ranker for 50 iterations.

We have also tested logistic regression optimized using liblinear, Long Short-Term Memories, Neural Networks, Passive Agressive classifiers, Random Forests, as well as Support Vector Machines with linear, polynomial and Radial Basis Function kernels. Logistic regression trained using Stochastic Gradient Descent is fast to train, does not require feature selection or dimension reduction and performs as well as or better than all other methods we have tested.

**Figure 4:**
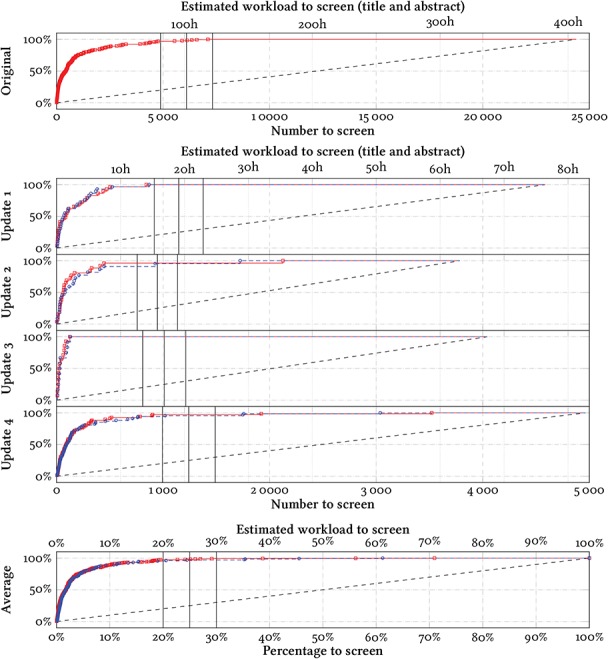
Effort-recall curves evaluating the system performance on the references with abstracts in each review update. The total number of data points are given in the leftmost column for each update in Table 1. The marks denote the positions in the ranking at which the included references would have been identified with screening prioritization, evaluated prospectively (blue circles) or retrospectively using cross-validation (red squares). The y-axes denote the percentage of identified included references (recall) throughout the screening process. The dashed lines denote the mean expected curve when screening in random order (equivalent to standard practice). We mark three hypothetical cut-offs at 20%, 25% and 30%. For scale, we give an estimate of the workload required by an experienced screener (one abstract in 1 minute). Inexperienced screeners may take longer, and we estimate fulltext screening to take $\sim $10 times longer than screening titles and abstracts.

We have observed no performance gains by using pairwise training over pointwise training.

**Figure 5:**
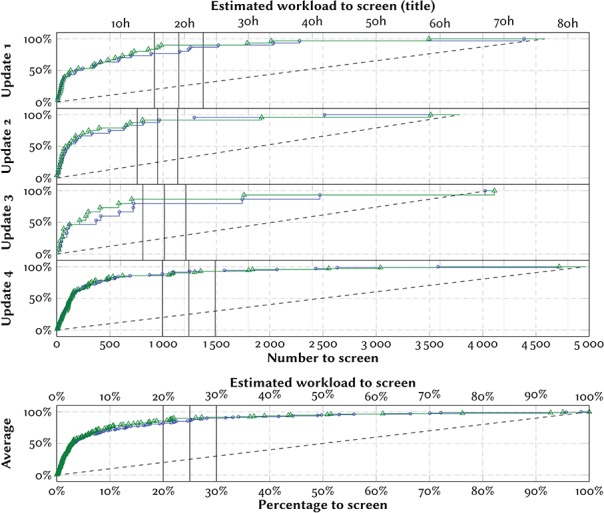
Effort-recall curves evaluating the system performance on titles only. The marks denote the positions in the ranking at which the included references would have been identified with screening prioritization, trained and evaluated only on titles (blue circles) or trained on titles and abstract and evaluated on titles (green triangles). The y-axes denote the percentage of identified included references (recall) throughout the screening process. The dashed lines denote the mean expected curve when screening in random order (equivalent to standard practice). We mark three hypothetical cut-offs at 20%, 25% and 30%. For scale, we give an estimate of the workload required by an experienced screener (one title in 1 min). We estimate that the time required to screen titles is on average the same as screening abstracts.

To compensate for the imbalance between the number of positive and negative references we increased the training weight for the positive examples to 80. Furthermore, we performed logistic regression with *L*_2_ regularization using $\alpha = 10^{-4}$. Each of these parameter settings was chosen as good default values in experiments on systematic reviews of drug class efficacy, and has proved to generalize well to systematic reviews of diagnostic test accuracy. Unlike in our previous work, we did not use under- or oversampling to compensate for the imbalance, because our previous experiments suggest this has limited benefit when used in addition to adjusting the training weights, and that the amount of under- or oversampling is often difficult to tune. We used default settings for all other parameters.

### Evaluation

We evaluate in terms of observed trade-off between effort and recall (sensitivity). We define effort as the absolute number of articles screened manually by the human screeners:

$$\begin{equation*} \textrm{effort} = \textrm{TP} + \textrm{FP} \end{equation*}$$

where TP denotes the true positives, and FP denotes the false positives.

We define recall as the proportion of positives (relevant articles) that are correctly identified:

$$\begin{align*} \textrm{recall} = \textrm{sensitivity} = \frac{\textrm{TP}}{\textrm{TP} + \textrm{FN}} \end{align*}$$

where TP denotes the true positives, and FN denotes the false negatives.

The effort and recall are positively correlated, and vary as the cut-off value is varied. Similarly to e.g. ROC curves, we will plot pairs of effort/recall value pairs over all possible cut-offs to simplify the selection of an appropriate trade-off between effort and recall.

## Results

We report the results of our experiments as effort-recall curves in Figures 4 and 5.

In the simulated prospective evaluation, we would have found the last included reference at position 870/4587 in update 1 (19.0%), position 1723/3785 in update 2 (45.5%), position 131/4043 in update 3 (3.4%) and position 3038/4963 in update 4 (61.2%) (Figure 4). Accepting some losses in update 4, we could have identified 67/68 references (98.5%) at position 1758 (35.4%), 66/68 references (97%) at position 1748 (35.2%) or 65/68 references (95.6%) at position 1020 (20.6%). The last two references in update 2 appear to be outliers, and we would have identified 21/22 references (95.5%) at position 926 (18.7%), or 20/22 references (90.1%) at position 447 (9.0%).

If we had used this system and had stopped after screening 25% of the candidate references, we would have identified 126 out of the 129 deduplicated references with abstracts in the four review updates (97.7%) (Figure 4).

In the simulated retrospective evaluation (using cross-validation), we would have found the last included reference at position 7102/24 384 in the original review (29.1%), position 843/4587 in update 1 (18.4%), position 2125/3785 in update 2 (56.1%), position 125/4043 in update 3 (3.1%) and position 3521/4963 in update 4 (70.9%) (Figure 4). Accepting some losses in update 4, we could have identified 67/68 references (98.5%) at position 1921 (38.7%), 66/68 references (97%) at position 902 (18.2%). Similarly to the prospective evaluation results, the last reference in update 2 appears to be an outlier, and we would have identified 21/22 references (95.5%) at position 446 (9.0%).

Overall, there was only a small difference between the prospective and the retrospective results, and the retrospective results were consistently better only in update 2 (Figure 4). Stopping after screening 25% of the candidate references in the retrospective evaluation would have identified 317 out of the 324 deduplicated references with abstracts in the four review updates (97.7%) (Figure 4).

In the dataset, 3840 out of 45 602 articles lacked abstracts (8.4%), of which 8 were included in the systematic review. The performance of the model was unreliable when evaluated on these. The area under the curves were visibly lower (Figure 5). Correspondingly, stopping after screening 25% of references would only have identified 2/6 references in the prospective evaluation, and 22/29 references (75.9%) in the retrospective evaluation.

The model performed substantially worse when evaluating on only titles (Figure 5). A model trained using all prior references, but trained and evaluated only on titles would on average have identified 86% of the relevant references after screening 25% of the candidates (Figure 5, bottom). A model trained on titles and abstracts from all prior references, but evaluated only on titles would on average have identified 90% of the relevant references after screening 25% of the candidates (Figure 5, bottom). Using a more conservative threshold would not have helped—several of the relevant references were identified only at the end of the simulated screening.

However, only 3840 out of 45 602 references in the dataset lacked abstracts (8.4%). These references constitute <300 references in each review, corresponding to a workload of <5 hours of screening per reviewer.

## Discussion

We used a logistic regression model for automatic article ranking to assess the suitability of automated screening for future updates to an annual systematic review of COS. We estimate that this model of automatic ranking can decrease the number of references that need to be screened by 75% while identifying $\sim $98% of all relevant references on average.

The results of this study are encouraging, and suggest that automated screening can be used to reduce the workload and therefore time and cost associated with this annual update. While we anticipate a reduction of workload by 75% and 62.5 hours per screener in the abstract screening stage, a balance needs to be struck with the prospect of identifying all eligible studies. With the last included reference identified at position 3038 in the previous update, it is realistic to accept that all studies might not be identified using this ranking method if a reduction in time and workload is desired. However, 97.8% of articles (317/324) could still be identified retrospectively and 97.7% of articles (126/129) could be identified prospectively if a different position was selected for the cut-off point for screening. Other methods of identifying relevant studies are employed in the update of the systematic review of COS, such as hand-searching, reference checking, relevant database alerts for key words and references, as well as checking with known experts. These other methods of identifying relevant papers increase the likelihood that all eligible studies will continue to be identified, and mean that a balance can be struck between managing the workload and identifying all eligible studies.

The results of this study showed that the screening automation can be reliable, provided both titles and abstracts are available, but that the automated ranking cannot reliably identify included references based only on titles. However, the number of references without abstracts is relatively low and we estimate that screening these manually would only take 2–4 hours per screener. We therefore recommend these be screened manually also in future updates of the systematic review of COS.

## Conclusions

Based on the results in this study we determined that stopping after screening the first 25% of the candidate studies would result in a loss of roughly 2% of the relevant studies, which we deemed an acceptable trade-off in this systematic review. However, the same stopping criterion would have resulted in a loss of over 10% of the relevant studies without abstracts. Balancing the risk of missing relevant references against the limited number of such references, we opted to screen all references without abstracts manually.

We are currently using this system based on logistic regression to identify Core Outcome Sets published in 2018 for the fifth update of the Comet database. The database searches were performed in March 2019 and the screening is currently ongoing. The prospective use of these methods will further validate these results and this model of automated screening. This study has demonstrated that automation has great potential to make the annual updates of this systematic review quicker and cheaper.

## Notes

**Database URL**: http://www.comet-initiative.org.
